# The nonlinear relationship between thyroid function parameters and metabolic dysfunction-associated fatty liver disease

**DOI:** 10.3389/fendo.2023.1115354

**Published:** 2023-02-22

**Authors:** Yingying Hu, Fan Zhou, Fang Lei, Lijin Lin, Xuewei Huang, Tao Sun, Weifang Liu, Xingyuan Zhang, Jingjing Cai, Zhi-Gang She, Hongliang Li

**Affiliations:** ^1^ Department of Cardiology, Renmin Hospital of Wuhan University, Wuhan, China; ^2^ Institute of Model Animal, Wuhan University, Wuhan, China; ^3^ Department of Gastroenterology, Huanggang Central Hospital of Yangtze University, Huanggang, China; ^4^ Huanggang Institute of Translational Medicine, Huanggang, China; ^5^ School of Basic Medical Science, Wuhan University, Wuhan, China; ^6^ Department of Cardiology, The Third Xiangya Hospital, Central South University, Changsha, China

**Keywords:** metabolic dysfunction-associated fatty liver disease, thyroid function parameters, nonlinear relationship, restricted cubic spline analysis, multivariable-adjusted logistic regression models

## Abstract

**Background:**

The relationship between thyroid function parameters and metabolic dysfunction-associated fatty liver disease (MAFLD) remains controversial. Additionally, little is known about the relationship between thyroid function parameters and MAFLD in the Chinese population.

**Methods:**

We conducted a retrospective cross-sectional study involving 177,540 individuals with thyroid function tests and MAFLD diagnosis from 2010-2018. The association between thyroid function parameters and MAFLD was evaluated on a continuous scale with restricted cubic spline (RCS) models and by the prior-defined centile categories with multivariable-adjusted logistic regression models. Thyroid function parameters included free triiodothyronine (FT3), free tetra-iodothyronine (FT4), and thyroid stimulating hormone (TSH). Additionally, fully adjusted RCS models stratified by sex, age, and location were studied.

**Results:**

In the RCS models, the risk of MAFLD increased with higher levels of FT3 when FT3 <5.58pmol/L, while the risk of MAFLD decreased with higher levels of FT3 when FT3 ≥5.58pmol/L (P nonlinearity <0.05). While RCS analysis suggested that the FT4 levels had a negative association with MAFLD (P nonlinearity <0.05), indicating an increase in FT4 levels was associated with a decreased risk of MAFLD. RCS analysis suggested an overall positive association between the concentration of TSH and MAFLD risk (P nonlinearity <0.05). The rising slope was sharper when the TSH concentration was less than 1.79uIU/mL, which indicated the association between TSH and MAFLD risk was tightly interrelated within this range. The multivariable logistic regression showed that populations in the 81st-95th centile had the highest risk of MAFLD among all centiles of FT3/TSH, with the 1st-5th centile as the reference category.

**Conclusions:**

Our study suggested nonlinear relationships between thyroid function parameters and MAFLD. Thyroid function parameters could be additional modifiable risk factors apart from the proven risk factors to steer new avenues regarding MAFLD prevention and treatment.

## 1 Introduction

Metabolic dysfunction-associated fatty liver disease (MAFLD) is a more inclusive term than nonalcoholic fatty liver disease (NAFLD) for the patient with associated metabolic dysfunction of hepatic steatosis (1). Although there is a substantial overlap between the two populations defined by MAFLD and NAFLD, the considerable differences in the two populations should not be neglected. Moreover, those populations diagnosed with MAFLD would have more comorbidities and worse prognoses than those with NAFLD (2, 3). Due to its clinically occult symptoms in the early stages, MAFLD often leads to severe outcomes, such as steatohepatitis, liver fibrosis, and even cirrhosis and hepatic carcinoma (4). Therefore, improving the awareness and management of its related risk factors could promote early screening to achieve early interventions.

The increasing prevalence and associated burden of MAFLD cannot be fully explained by traditional risk factors, such as age, gender, smoking, body mass index (BMI), hypertension, hyperlipidemia, and diabetes mellitus. Accumulating evidence has suggested that thyroid function parameters may also be associated with MAFLD (5). Physiologically, thyroid hormone (TH) powerfully influences metabolic processes through multiple pathways for human beings, with profound effects on lipid metabolism and energy expenditure (6). Their imbalanced levels may also lead to various unfavorable effects and contribute to the development and progression of multiple diseases, including MAFLD (7). Their importance promotes researchers exploring the relationship between thyroid function parameters and MAFLD. The conclusions from studies regarding the relationship between thyroid function parameters and NAFLD/MAFLD are inconsistent, varying from a solid association to no association (8–12). There are limited studies aiming to assess the association of thyroid function parameters with MAFLD in the large-scale population in China. In addition, the relationship between thyroid function parameters and MAFLD risk may not be linear, and prior studies have been relatively underpowered to assess nonlinear relationships. Therefore, we conducted a large retrospective study to evaluate the potential connection between the level of each thyroid function parameter and MAFLD risk and to further explore these relationships in different sex, age, and location groups.

## 2 Methods

### 2.1 Study population

**Figure 1:**
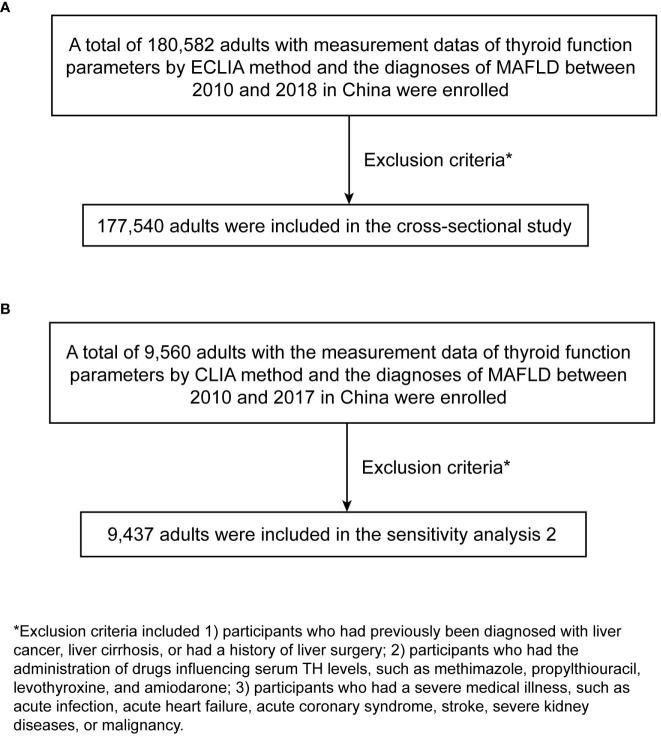
The flow chart of participant selection. **(A)** main study. **(B)** sensitivity analysis 2. ECLIA, electrochemiluminescence immunoassay; MAFLD, metabolic dysfunction-associated fatty liver disease; CLIA, chemiluminescence analysis.

This study was designed as a multicenter retrospective study comprising 180,582 adults from 5 health check-up centers from 2010 to 2018. All individuals had access to serum FT3, FT4, and TSH concentrations, as well as MAFLD diagnosis. Moreover, there were 3 centers in the north of China and 2 in the south of China. Participants who 1) had previously been diagnosed with liver cancer, liver cirrhosis, or had a history of liver surgery (N=227); 2) had the administration of drugs influencing serum TH levels, such as methimazole, propylthiouracil, levothyroxine, and amiodarone (N=24); 3) had a severe medical illness, such as acute infection, acute heart failure, acute coronary syndrome, stroke, severe kidney diseases, and malignancy (N=2791) were excluded. Finally, 177540 adults were enrolled in our multicentered retrospective study. The flow chart of participant selection is shown in **Figure 1A**.

This study was approved by the ethical review committee of Renmin Hospital of Wuhan University and followed by acceptance by the ethics center in each collaborating hospital. The ethics committees granted a waiver of the requirement for documentation of informed consent for just analyzing existing data after anonymization without individual identification.

### 2.2 Anthropometric and laboratory data

The medical histories were collected face-to-face and recorded by professional physicians, from which the demographic information of participants was extracted, including sex, age, smoking status, alcohol consumption, medications, medical history, and so on. All participants had undergone comprehensive anthropometric measurements and clinical examinations.

Anthropometric measurements, including height, weight, and waist circumference (WC), and physiological parameters, such as systolic and diastolic blood pressure (SBP and DBP), and heart rates, were performed by well-trained physicians according to standard protocols. BMI was calculated as weight divided by the square of height (kg/m^2^). After overnight fasting, the participants underwent clinical examinations, including routine blood examinations, biochemical tests, and liver ultrasounds. Biochemical tests included liver function tests, renal function tests, fasting blood glucose (FBG) levels, lipid contents examinations, and thyroid function tests. The estimated glomerular filtration rate (eGFR) was calculated by the Modification of Diet in Renal Disease equations (13).

All check-up centers included in our main study applied identical methodology, namely, the electrochemiluminescence immunoassay (ECLIA) method, to complete thyroid function tests. According to the ECLIA method, the overall ranges of thyroid function parameters were as follows: FT3 ranged from 0.39 to 76pmol/L (reference range, 3.43-6.5pmol/L), FT4 ranged from 0.3 to 294.2pmol/L (reference range, 12-22pmol/L), and TSH ranged from 0 to 230uIU/mL (reference range, 0.27-4.2uIU/mL). All imagological diagnoses were performed and evaluated by experienced imaging specialists at medical health check-up centers.

### 2.3 Diagnostic criteria

MAFLD was defined by evidence of hepatic steatosis on abdominal ultrasound, Computed Tomography, or Magnetic Resonance Imaging with the presence of one of the following three criteria: overweight or obesity (defined as BMI ≥23 kg/m^2^ in Asians); the presence of type 2 diabetes (T2DM); lean or normal weight (BMI <23 kg/m^2^) with the presence of or metabolic dysregulation. Metabolic dysregulation was defined by the presence of at least two of the following metabolic risk abnormalities: 1) WC ≥90 cm for men and 80 cm for women; 2) blood pressure ≥130/85 mmHg or on specific drug treatment; 3) plasma triglycerides ≥1.70 mmol/L or on specific drug treatment; 4) plasma high-density lipoprotein cholesterol (HDL-C) <1.0 mmol/L for men and <1.3 mmol/L for women or on specific drug treatment; 5) prediabetes (i.e., FBG was 5.6 to 6.9 mmol/L, or 2 hours postprandial glucose level was 7.8 to 11.0 mmol or glycosylated hemoglobin A1c level was 5.7% to 6.4%) (1). T2DM was diagnosed according to the clinical guidelines for the prevention and treatment of T2DM in the elderly in China (2022 edition) (14). Hypertension was defined as an SBP ≥140 mmHg and/or DBP ≥90 mmHg, a medical history of hypertension, or the use of antihypertensive agents according to the 2018 Chinese hypertension management guidelines (15). Metabolic syndrome (MetS) was defined based on the criteria within the CHPSNE (Control Hypertension and Other Risk Factors to Prevent Stroke with Nutrition Education in Urban Area of Northeast China) study (16).

### 2.4 Statistical analysis

The basic characteristics of participants were presented by descriptive statistics. The Kolmogorov–Smirnov test was used to evaluate the normality of the distribution of the continuous variables. Continuous variables were summarized as mean and standard deviation (SD) if normally distributed and median and interquartile range (IQR) if not normally distributed. Categorical variables were presented as frequencies and percentages. Student’s t-tests (normally distributed) and Wilcoxon rank-sum test (non-normally distributed) for continuous variables, and Fisher’s exact test or chi-square test for categorical variables were used to compare the intergroup differences.

Apart from dividing populations into with or without MAFLD groups, five equally distributed categories were defined by the 20th, 40th, 60th, and 80th centiles of the level of thyroid function parameters, and to evaluate the highest and lowest levels of thyroid function parameters, two additional categories were defined by the 5th and 95th centiles.

We used RCS models fitted for the logistic regression model to assess the potential nonlinear relationships between levels of thyroid function parameters on a continuous scale and MAFLD. To balance best fit and overfitting in the main splines for MAFLD, the number of knots, between three and five, was chosen as the lowest value for the Akaike information criterion, but if within two of each other for different knots, the lowest number of knots was determined. Then, analysis of variance was used to complete nonlinear tests. The concentration of thyroid function parameters associated with the highest risk of MAFLD was the concentration with the highest odds ratio (OR) on the spline curve. Analyses were adjusted for multiple variables. We considered the clinical significance, the baseline difference, and the results of previous studies to determine the adjusted variables (17–20), including age, sex, heart rates, leukocyte counts, red blood cells, platelet counts, hemoglobin, gamma-glutamyl transpeptidase, eGFR, uric acid, total cholesterol, smoking status and alcohol consumption, a history of DM and hypertension. Moreover, the multivariable stratification analysis was conducted according to gender, age, and location. The same number of knots from the main splines was also applied in splines for stratified analysis to allow a direct comparison of overall and stratified analyses. Furthermore, the associations between seven predefined TH categories and MAFLD were examined. Multivariable logistic regression models adjusting for covariates mentioned above were applied to estimate OR and 95% confidence intervals (CI) for MAFLD. The reference category for these analyses was the lowest level of thyroid function parameters.

The nonparametric missing value imputation based on the missForest procedure in R was used to fill in the missing data. The results of the subsequent analysis of the dataset before and after imputation were not significantly different.

R software (version 4.1.0) was used to perform all statistical analyses and create all graphs; a two‐sided P <0.05 was considered statistically significant.

### 2.5 Sensitivity analysis

We further conducted two sensitivity analyses. In our main analysis, BMI was not adjusted because it belongs to one of the diagnostic criteria of MAFLD. While in sensitivity analysis 1, we further adjusted BMI besides covariates in the main analysis, given the possibility that it could be a confounder. In sensitivity analysis 2, populations used the chemiluminescence analysis (CLIA) method to detect thyroid function parameters, which is an entirely different detection method from ECLIA. There is a large difference between the reference ranges of the two methods, and the ECLIA has the characteristics of higher sensitivity, specificity, and selectivity than CLIA. Finally, there were 9560 participants aged ≥18 years enrolled in sensitivity analysis 2 to explore the relationship between thyroid function parameters and MAFLD. They came from 3 health check-up centers covering 3 administrative regions between 2010 and 2017 in China. They all underwent the detection of thyroid function parameters and had the diagnosis of MAFLD. Then based on the exclusion criteria mentioned above, 123 participants were excluded. Finally, 9437 adults were for sensitivity analysis 2. The flow chart of participant selection is shown in **Figure 1B**. Sensitivity analysis 2 was performed using a statistical method similar to the main analysis.

## 3 Results

### 3.1 Clinical and laboratory characteristics

The main analysis consisted of 177,540 participants with a median age of 48 (IQR, 42, 54) and 60.19% males. The baseline characteristics of participants are summarized in **Table 1**. In the overall population, 51.14% of participants had MAFLD. Compared with the participants without MAFLD, those with MAFLD tended to be older, to be male; to have higher BMI, thicker WC, higher SBP and DBP, more leukocyte counts and red blood cells, higher hemoglobin, alanine aminotransferase, aspartate transaminase, gamma-glutamyl transpeptidase, serum creatinine, blood urea nitrogen, uric acid, FBG, total cholesterol, triglycerides, and low-density lipoprotein cholesterol; fewer platelet counts and lower HDL-C; more likely to be smokers and drinkers, and to be more likely have hypertension, diabetes, and dyslipidemia.

**Table 1:** Clinical and laboratory characteristics of participants with and without MAFLD from 2010 to 2018.

Characteristics	Total (N = 177540)	Non-MAFLD (N = 86753, 48.86%)	MAFLD (N = 90787, 51.14%)	P-value
Clinical characteristics				
Age (years, median [IQR])	48.00 [42.00, 54.00]	46.00 [39.00, 53.00]	49.00 [43.00, 54.00]	<0.05
Gender, Male, n (%)	106859 (60.19)	37607 (43.35)	69252 (76.28)	<0.05
BMI (kg/m^2^, median [IQR])	24.76 [22.48, 27.04]	22.47 [20.77, 24.28]	26.48 [24.81, 28.43]	<0.05
WC (cm, median [IQR])	87.00 [79.00, 94.00]	79.00 [73.00, 86.00]	93.00 [87.00, 98.00]	<0.05
Self-reported smoking, n (%)	26119 (14.71)	8201 (9.45)	17918 (19.74)	<0.05
Self-reported drinking, n (%)	39846 (22.44)	13462 (15.52)	26384 (29.06)	<0.05
SBP (mmHg, median [IQR])	120.00 [109.00, 132.00]	114.00 [104.00, 126.00]	126.00 [115.00, 137.00]	<0.05
DBP (mmHg, median [IQR])	78.00 [70.00, 87.00]	74.00 [67.00, 81.00]	82.00 [75.00, 90.00]	<0.05
Laboratory Examination				
Heart rates (/min, median [IQR])	70.00 [64.00, 76.00]	70.00 [64.00, 75.00]	70.00 [65.00, 76.00]	<0.05
LEU (×10^9^/L, median [IQR])	5.82 [4.94, 6.88]	5.56 [4.71, 6.59]	6.06 [5.20, 7.11]	<0.05
RBC (×10^12^/L, median [IQR])	4.79 [4.46, 5.12]	4.62 [4.32, 4.96]	4.93 [4.63, 5.21]	<0.05
PLT (×10^9^/L, median [IQR])	220.00 [188.00, 257.00]	222.00 [189.00, 258.00]	219.00 [186.00, 255.00]	<0.05
HGB (g/L, median [IQR])	147.00 [135.00, 158.00]	140.00 [130.00, 152.00]	152.00 [142.00, 161.00]	<0.05
ALT (IU/L, median [IQR])	19.40 [13.80, 28.70]	15.70 [11.70, 22.00]	23.80 [17.10, 34.40]	<0.05
AST (IU/L, median [IQR])	18.40 [15.50, 22.60]	17.40 [14.90, 21.00]	19.50 [16.30, 24.20]	<0.05
GGT (IU/L, median [IQR])	26.00 [16.00, 46.00]	17.90 [12.40, 29.00]	35.10 [23.00, 59.00]	<0.05
Scr (μmol/L, median [IQR])	68.00 [57.60, 78.00]	64.00 [55.00, 75.10]	71.00 [61.00, 80.00]	<0.05
BUN (mmol/L, median [IQR])	4.90 [4.11, 5.72]	4.70 [3.92, 5.53]	5.06 [4.30, 5.90]	<0.05
UA (μmol/L, median [IQR])	327.00 [264.40, 391.70]	287.00 [238.00, 349.00]	362.00 [305.70, 419.50]	<0.05
FBG (mmol/L, median [IQR])	5.32 [4.97, 5.82]	5.12 [4.83, 5.47]	5.56 [5.17, 6.21]	<0.05
TC (mmol/L, median [IQR])	4.72 [4.15, 5.35]	4.62 [4.06, 5.22]	4.83 [4.24, 5.46]	<0.05
TG (mmol/L, median [IQR])	1.37 [0.94, 2.05]	1.05 [0.77, 1.48]	1.76 [1.26, 2.54]	<0.05
LDL-C (mmol/L, median [IQR])	3.00 [2.47, 3.55]	2.87 [2.37, 3.41]	3.12 [2.58, 3.66]	<0.05
HDL-C (mmol/L, median [IQR])	1.24 [1.03, 1.49]	1.39 [1.17, 1.65]	1.12 [0.95, 1.31]	<0.05
FT3 (pmol/L, median [IQR])	4.90 [4.48, 5.34]	4.77 [4.36, 5.22]	5.01 [4.61, 5.44]	<0.05
FT4 (pmol/L, median [IQR])	16.24 [14.80, 17.80]	16.22 [14.77, 17.82]	16.26 [14.84, 17.78]	<0.05
TSH (uIU/mL, median [IQR])	2.07 [1.42, 3.02]	2.10 [1.42, 3.09]	2.04 [1.42, 2.96]	<0.05
Comorbidities				
Type 2 diabetes, n (%)	23349 (13.19)	4151 (4.81)	19198 (21.16)	<0.05
Hypertension, n (%)	44234 (26.27)	11347 (14.42)	32887 (36.66)	<0.05
MetS, n (%)	82226 (48.46)	17423 (21.84)	64803 (72.07)	<0.05

MAFLD, metabolic dysfunction-associated fatty liver disease; IQR, interquartile range; BMI, body mass index; WC, waist circumference; SBP, systolic blood pressure; DBP, diastolic blood pressure; LEU, leukocyte counts; RBC, red blood cells; PLT, platelet counts; HGB, hemoglobin; ALT, alanine aminotransferase; AST, aspartate transaminase; GGT, gamma-glutamyl transpeptidase; Scr, serum creatinine; BUN, blood urea nitrogen; UA, uric acid; FBG, fasting blood glucose; TC, total cholesterol; TG, triglycerides; LDL-C, low-density lipoprotein cholesterol; HDL-C, high-density lipoprotein cholesterol; FT3, free triiodothyronine; FT4, free tetraiodothyronine; TSH, thyroid-stimulating hormone; MetS, metabolic syndrome.

### 3.2 Association between FT3 levels on a continuous scale and MAFLD

**Figure 2:**
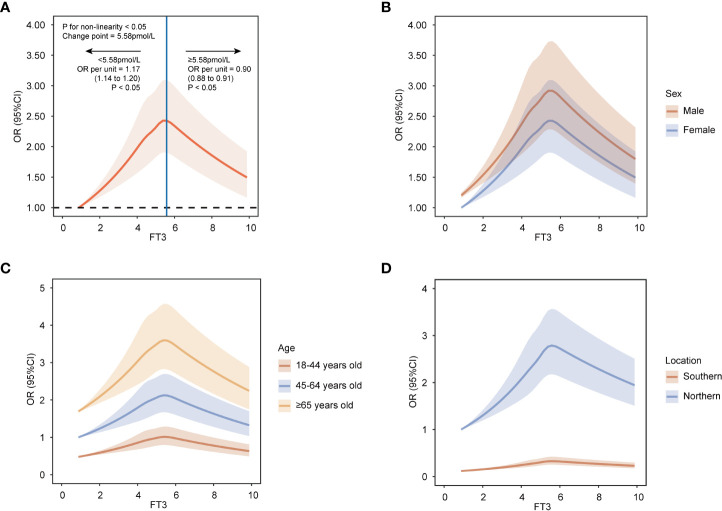
Restricted cubic spline analyses with five knots for nonlinear association between FT3 levels and MAFLD on a continuous scale. **(A)** all population. **(B)** by sex. **(C)** by age groups. **(D)** by location groups. ORs are indicated by solid lines and 95% CIs by shaded areas. Reference point is lowest value for FT3. Analyses were adjusted for age, sex, heart rates, leukocyte counts, red blood cells, platelets, hemoglobin, gamma-glutamyl transpeptidase, estimated glomerular filtration rate, uric acid, total cholesterol, smoking status, alcohol consumption, and a history of diabetes and hypertension. FT3, free triiodothyronine; MAFLD, metabolic dysfunction-associated fatty liver disease; OR, odd ratio; CI, confidence interval.

**Table S1** shows the baseline characteristics of participants according to prior-defined centile categories of FT3. To assess the nonlinear relationship, the association between FT3 and MAFLD was estimated on a continuous scale using RCS. In the RCS models, the risk of MAFLD increased with the levels of FT3 when FT3 concentration was less than 5.58pmol/L, while the risk of MAFLD decreased with the levels of FT3 when FT3 concentration was more than or equal to 5.58pmol/L (P nonlinearity < 0.05) (**Figure 2A**). The lowest and highest levels of FT3 were associated with a decreased risk of MAFLD.

**Figure 3:**
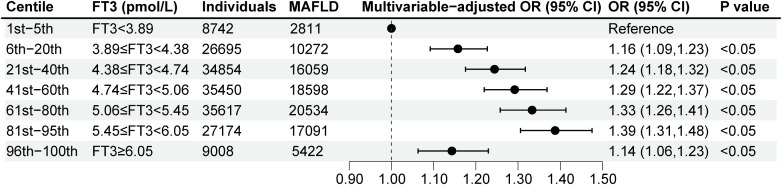
Multivariable-adjusted logistic regression analyses for MAFLD according to FT3 levels by the prior-defined centile categories. Analyses were adjusted for age, sex, heart rates, leukocyte counts, red blood cells, platelets, hemoglobin, gamma-glutamyl transpeptidase, estimated glomerular filtration rate, uric acid, total cholesterol, smoking status, alcohol consumption, and a history of diabetes and hypertension. FT3, free triiodothyronine; MAFLD, metabolic dysfunction-associated fatty liver disease; OR, odd ratio; CI, confidence interval.

To estimate precise OR values, the multivariable logistic regression model was used. When FT3 concentration was less than 5.58pmol/L, the OR per unit higher FT3 was 1.17 (95% CI, 1.14, 1.20) (**Figure 2A**), which indicated each unit increase in FT3 concentration was associated with a 0.17-fold increased risk of MAFLD. While when FT3 concentration was more than or equal to 5.58pmol/L, the OR per unit higher FT3 was less than 1 with being 0.90 (95% CI, 0.88, 0.91) (**Figure 2A**), suggesting the risk of MAFLD decreased by 0.1-fold for every unit increase of FT3 concentration. Moreover, according to the prior-defined centile categories, we calculated the OR of each centile of FT3 with the 1st-5th centile as a reference group. The highest multivariable-adjusted OR for MAFLD was 1.39 (95% CI, 1.31, 1.48) for individuals with FT3 concentrations of 5.45-6.05pmol/L (81st-95th centile) among all centiles (**Figure 3**). The population with the FT3 concentrations of 5.45-6.05pmol/L (81st-95th centile) had the highest risk of MAFLD.

We performed stratified analyses to assess possible effect modification according to sex, age, and location. The nonlinear relationship between FT3 and MAFLD was observed among various sex, age, and location groups (**Figures 2B–D**). Furthermore, the association was most pronounced in males, individuals over 65 years old, and in populations in Northern China among different subgroups (**Figures 2B–D**).

### 3.3 Association between FT4 levels on a continuous scale and MAFLD

We also explored the association between FT4 and MAFLD. Baseline characteristics of participants by prior-defined centile categories of FT4 are summarized in **Table S2**. RCS analysis suggested the FT4 levels on a continuous scale had a negative association with MAFLD (P nonlinearity <0.05) (**Figure S1A**), indicating an increase in FT4 levels was associated with a decreased risk of MAFLD.

After adjustment for identified covariates, the multivariable logistic regression model presented that OR per unit higher FT4 was 0.93 (95% CI, 0.93, 0.93) (**Figure S1A**), showing every unit increase of FT4 was associated with a 0.07-fold lower risk of MAFLD. In parallel, we found that individuals with concentrations of FT4 more than or equal to 20.43pmol/L (96th-100th centiles) had the lowest MAFLD risk among all centiles, with the 1st -5th centile being a reference group (OR, 0.45 [95% CI, 0.42, 0.48]) (**Figure S2**).

Results did not change substantially by further dividing people by sex, age, and location, and the declining relationship between FT4 levels and MAFLD persisted. Additionally, the most pronounced association was observed in males, individuals over 65 years old, and in populations in Northern China among different subgroups (**Figure S1B–D**).

### 3.4 Association between TSH levels on a continuous scale and MAFLD

We further explored the relationship between TSH and MAFLD. **Table S3** shows the baseline characteristics of participants according to prior-defined centile categories of TSH. RCS analysis suggested an overall positive association between the concentration of TSH and MAFLD risk (P nonlinearity <0.05) (**Figure S3A**). The rising slope was sharper when the TSH concentration was less than 1.79uIU/mL, which indicated the association between TSH and MAFLD risk was tightly interrelated within this range.

For individuals whose TSH level was less than 1.79uIU/mL, the OR per unit higher TSH was 1.28 (95% CI, 1.22, 1.33) (**Figure S3A**), which indicated each unit increase in TSH concentration was associated with a 0.28-fold increased risk of MAFLD. For individuals whose TSH level was more than or equal to 1.79uIU/mL, the 95% confidence interval included the OR of 1.00 (OR, 1.00 [95% CI, 1.00, 1.00], P value =0.42) (**Figure S3A**), suggesting the risk of MAFLD slightly increased with each unit increase in TSH concentration, but this trend was not statistically significant. At the same time, with the 1st-5th centile as the reference category, the highest multivariable-adjusted OR for MAFLD was 1.40 (95% CI, 1.33, 1.48) for individuals with TSH concentrations of 3.33-5.60uIU/mL (81st-95th centile) (**Figure S4**). The individuals with the TSH concentrations of 3.33-5.60uIU/mL (81st-95th centile) had the highest risk of MAFLD.

The association between TSH concentrations and MAFLD was not significantly modified with respect to sex, age, and location. Moreover, TSH also showed a stronger association with rising shape within a certain range with MAFLD in males, individuals over 65 years old, and individuals in Northern China than in outer subgroups (**Figure S3B–D**).

### 3.5 Sensitivity analysis

To validate the robustness of our results, we conducted two sensitivity analyses. The results of sensitivity analyses did not change substantially. In sensitivity analysis 1, in addition to covariates in the main analysis, BMI was regarded as a confounder. The shape with a first rising and then decline trend between FT3 and MAFLD and the shape with a rising trend in a certain range between TSH and MAFLD persisted (P nonlinearity <0.05) (**Figures S5A, S5C**). In **Figure S5B**, for FT4, the overall declining trend was observed, although the curve has changed a bit (BMI was regarded as an important confounder) (**Figure S5B**). In sensitivity analysis 2, the data from various health check-up centers and different detection methods were used to explore the association between thyroid function parameters on a continuous scale and MAFLD. The baseline characteristics of participants in sensitivity analysis 2 are summarized in **Table S4**. The association between the concentrations of variations in thyroid function and MAFLD was not significantly modified by health check-up centers and detection methods (**Figure S6A–C**).

## 4 Discussion

This large population-based study among general individuals demonstrated nonlinear relationships between thyroid function parameters and MAFLD. The populations with FT3 in the 81st-95th centile level, FT4 in the 1st-5th centile level, and TSH in the 81st-95th centile level were most likely to develop MAFLD among all centiles. Different stratifications according to gender, age, and location influenced the strength of the nonlinear relationships between thyroid function parameters and MAFLD. Taken together, thyroid function parameters could be additional modifiable risk factors apart from the proven risk factors to steer new avenues regarding MAFLD prevention and treatment.

Because there was a substantial overlap between the MAFLD and NAFLD, our observations between thyroid function parameters and MAFLD could be comparable with the findings between thyroid function parameters and NAFLD. In our results, the population could be divided into two groups based on the relationship between FT3 and MAFLD risk: group one with FT3 less than 5.58pmol/L (within the reference range), where MAFLD risk increased with higher FT3; group two with FT3 more than or equal to 5.58pmol/L, where MAFLD risk decreased with higher FT3. Other studies on thyroid function and NAFLD have concentrated on three subtypes: individuals with hypothyroidism (12, 21), euthyroid subjects (8, 9, 11, 22–25), and individuals with hyperthyroidism (26). The diagnosis of hypothyroidism does not involve FT3 (27). Notably, most studies involving euthyroid subjects have concluded that higher FT3 is associated with an increased risk of NAFLD (8, 11, 22, 23). However, these studies divided the FT3 levels into multiple quartiles and did not investigate its association with MAFLD on a continuous range. However, others did not find a positive association (9, 24, 25). Comparison with results from various studies is different because of varying population selection and experimental design. In addition, our findings implied that population distribution before and after the change point significantly impacts the study results. In the study of hyperthyroid patients, elevated FT3 is associated with a reduced risk of MAFLD. Therefore, cohort studies are needed to prove the relationship between FT3 and MAFLD on a continuous scale.

FT3 is commonly acknowledged to be more biologically active as a modulator of metabolic processes than FT4 (28). As a result, from a pathophysiological perspective, the relationship of MAFLD with FT3 rather than with FT4 should be regarded as the most relevant. Our findings could be explained by the significant contribution of TH to hepatic lipid metabolism. Cell studies have shown that TH stimulates lipolysis to generate circulating free fatty acids (FFAs), which are the major source of lipids for the liver. Then, TH promotes FFAs uptake to regulate lipid metabolism (29). In addition to promoting the uptake of exogenous FFAs, TH can also promote de novel lipogenesis in the liver stimulated by excess glucose directly and indirectly (30). Moreover, FT3 promotes triacylglycerol stored as lipid droplets in the liver to be hydrolyzed back to FFAs *via* classic lipases and lipophagy. Afterward, FFAs are broken down, undergoing mitochondrial β-oxidation to produce energy, which also is promoted by FT3 (31). Taken together, TH maintains the balance between lipid metabolism by stimulation of lipid synthesis and lipid oxidation by direct and indirect actions (5, 32). Combining this mechanism with the results of our study, it appears that when FT3 is great than 5.58pmol/L (81st-95th centile), more enhanced lipid oxidation than lipid synthesis exists.

Retrospective studies could not explore a causal relationship between elevated FT3 and increased risk of MAFLD. The current studies suggest that hypothyroidism increases the risk of NAFLD (12, 33). We suggest that the positive relationship between FT3 concentrations and MAFLD risk in group one (FT3 less than 5.58pmol/L) is due to obesity. The relationship between obesity and thyroid hormone levels that obese individuals have higher circulating FT3 has been elaborated in numerous investigations (34–37). In addition, Mendelian randomization research documented that higher BMI/fat mass is a determinant of increasing FT3 levels (36). The mechanisms responsible for this action are not yet precisely known, and several assumptions exist. Firstly, this action may involve tissue-specific alterations in iodothyronine deiodinase (DIO) expression in relation to obesity (38). Previous studies have suggested DIO 1 and/or 2 activities in subjects with a relatively higher fat mass and/or a less favorable metabolic profile would change, leading to a higher conversion of FT4 to FT3 (39–41). Secondly, obesity may alter the hypothalamic-pituitary-thyroid axis (42, 43). Thirdly, observed changes in FT3 may relate partly to excess carbohydrates in the diet of obese individuals (44). Furthermore, the importance of obesity in the pathogenesis of MAFLD is well established (45). The cross-sectional design of the present study hampers to establish the causation between FT3 and MAFLD, and the possible interrelationship of obesity with FT3 and the development of MAFLD needs to be prospectively delineated in the future.

Given the key role of TH on hepatic lipid accumulation, much effort has been recently paid to developing a liver-targeted agonist of THRβ, which has been shown to diminish hepatic lipid accumulation in animal studies (46–48). In addition, animal studies have shown that triiodothyronine administration caused a rapid regression of fully established steatosis (49). However, due to the side effects (especially in the heart, muscle, and bone), none of these drugs has been introduced into clinical practice, which underscores the complexity of TH physiology.

For TSH, inconsistent associations of serum TSH with NAFLD were reported with positive or null results among various populations and research (8, 9, 11, 24, 50–52). We must take the negative feedback loop between TSH and TH into consideration, namely, the production of TSH and TH is regulated by the hypothalamic–pituitary–thyroid axis. Specifically, TSH stimulates the thyroid to synthesize and release TH and in turn, TH acts on the pituitary and hypothalamus to inhibit TSH production. In addition to the interaction with thyroxine, the TSH upregulates the expression of hepatic 3-hydroxy-3-methyl-glutaryl coenzyme A reductase to promote the synthesis of cholesterol to direct effect on lipids and BMI (53–56). In addition, TSH can robustly stimulate the secretion of leptin to affect BMI (57, 58). In a word, TSH is a complex regulation process, which could explain the variations of the results of TSH.

Several limitations should be noted in our study. Firstly, there are inherent limitations in inferring the causal relationship between TH and MAFLD in our retrospective study. We cannot infer direct causes and effects between TH and MAFLD. Secondly, since MAFLD is mainly diagnosed by ultrasonography, we could not determine the severity of MAFLD-associated hepatitis and might not detect mild steatosis. However, up to now, ultrasonography is still a safe and confirmed reliable noninvasive method, compared with the pathological diagnosis by liver biopsy with higher diagnostic accuracy limited by its invasive, impractical and costly. Thirdly, limited information on smoking and drinking consumption status, medications history, and past medical history may result in bias due to an insufficient adjustment of these confounders in the models.

## 5 Conclusions

Our study suggested nonlinear relationships between thyroid function parameters and MAFLD. The populations with FT3 in the 81st-95th centile level, FT4 in the 1st-5th centile level, and TSH in the 81st-95th centile level were most likely to develop MAFLD among all centiles. Further mechanism research and large prospective studies with long-term follow-up are warranted to provide more definitive evidence to clarify the causal relationship between thyroid function parameters and MAFLD.

## Data availability statement

The datasets presented in this article are not readily available because privacy or ethical restrictions. The data that support the findings of this study are available on request from the corresponding author. Requests to access the datasets should be directed to Hongliang Li, lihl@whu.edu.cn.

## Ethics statement

The studies involving human participants were reviewed and approved by the ethical review committee of Renmin Hospital of Wuhan University and followed by acceptance by the ethics center in each collaborating hospital. The ethics committees granted a waiver of the requirement for documentation of informed consent for just analyzing existing data after anonymization without individual identification. Written informed consent for participation was not required for this study in accordance with the national legislation and the institutional requirements.

## Author contributions

YH and FZ designed the study, collected and analyzed data, and wrote the manuscript. FL, LL and XH collected and reviewed data and contributed to data analysis. TS, WL, XZ, and JC revised the manuscript and provided valuable suggestions for study design and data analysis. Z-GS and HL contributed equally, designed the project, edited the manuscript, and supervised the study. All authors have approved the final version of this paper.
