# Supplementary material for: The nonlinear relationship between thyroid function parameters and metabolic dysfunction-associated fatty liver disease
Source: Front Endocrinol (Lausanne). 2023 Feb 22;14:1115354. doi: 10.3389/fendo.2023.1115354 (PMC9992977; doi:10.3389/fendo.2023.1115354)
Supplement: Supplementary file 1 [file DataSheet_1.pdf]

Supplemental Material

Section 1: Table S1-S4

Table S1. Clinical and laboratory characteristics of participants according to prior-defined centile categories of FT3 from 2010 to 2018.

Table S2. Clinical and laboratory characteristics of participants according to prior-defined centile categories of FT4 from 2010 to 2018.

Table S3. Clinical and laboratory characteristics of participants according to prior-defined centile categories of TSH from 2010 to 2018.

Table S4. Clinical and laboratory characteristics of participants with and without MAFLD from 2010 to 2017 in sensitivity analysis 2.

Section 2: Figure S1-S6

Figure S1. Restricted cubic spline analyses with four knots for nonlinear association between FT4 levels and MAFLD on a continuous scale. (A) all population. (B) by sex. (C) by age groups. (D) by location groups.

Figure S2. Multivariable-adjusted logistic regression analyses for MAFLD according to FT4 levels by the prior-defined centile categories.

Figure S3. Restricted cubic spline analyses with five knots for nonlinear association between TSH levels and MAFLD on a continuous scale. (A) all population. (B) by sex. (C) by age groups. (D) by location groups.

Figure S4. Multivariable-adjusted logistic regression analyses for MAFLD according to TSH levels by the prior-defined centile categories.

Figure S5. Restricted cubic spline analyses with different knots for nonlinear association between thyroid function parameters and MAFLD on a continuous scale in sensitivity analysis 1. (A) FT3. (B) FT4. (C) TSH.

Figure S6. Restricted cubic spline analyses with different knots for nonlinear association between thyroid function parameters and MAFLD on a continuous scale in sensitivity analysis 2. (A) FT3. (B) FT4. (C) TSH.

Table S1. Clinical and laboratory characteristics of participants according to prior-defined centile categories of FT3 from 2010 to 2018.

Characteristics	All	Centile (pmol/L)							P-value
		1st-5th (FT3<3.89)	6th-20th (3.89≤FT3<4.38)	21st-40th (4.38≤FT3<4.74)	41st-60th (4.74≤FT3<5.06)	61st-80th (5.06≤FT3<5.45)	81st-95th (5.45≤FT3<6.05)	96st-100th (FT3≥6.05)	
	N = 177540	N = 8742	N = 26695	N = 34854	N = 35450	N = 35617	N = 27174	N = 9008	
**Clinical characteristics**									
Age (years, median [IQR])	48.00 [42.00, 54.00]	50.00 [44.00, 57.00]	49.00 [43.00, 55.00]	49.00 [43.00, 55.00]	48.00 [42.00, 54.00]	47.00 [41.00, 53.00]	46.00 [39.00, 51.00]	44.00 [36.00, 50.00]	<0.05
Gender, Male, n (%)	106859 (60.19)	2103 (24.06)	8991 (33.68)	16857 (48.36)	22035 (62.16)	26292 (73.82)	23011 (84.68)	7570 (84.04)	<0.05
BMI (kg/m^2, median [IQR])	24.76 [22.48, 27.04]	23.33 [21.25, 25.69]	23.75 [21.59, 26.08]	24.35 [22.10, 26.60]	24.83 [22.59, 27.05]	25.18 [22.99, 27.39]	25.58 [23.49, 27.80]	25.60 [23.33, 27.96]	<0.05
WC (cm, median [IQR])	87.00 [79.00, 94.00]	80.00 [73.00, 88.00]	82.00 [75.00, 90.00]	85.00 [77.00, 93.00]	88.00 [80.00, 94.00]	89.00 [82.00, 96.00]	91.00 [84.00, 97.00]	91.00 [84.00, 97.00]	<0.05
Self-reported smoking, n (%)	26119 (14.71)	327 (3.74)	1561 (5.85)	3529 (10.13)	5272 (14.87)	6803 (19.10)	6431 (23.67)	2196 (24.38)	<0.05
Self-reported drinking, n (%)	39846 (22.44)	715 (8.18)	3214 (12.04)	6174 (17.71)	8243 (23.25)	9872 (27.72)	8798 (32.38)	2830 (31.42)	<0.05
SBP (mmHg, median [IQR])	120.00 [109.00, 132.00]	114.00 [103.00, 128.00]	116.00 [104.00, 129.00]	118.00 [107.00, 131.00]	121.00 [109.00, 133.00]	122.00 [111.00, 133.00]	124.00 [113.00, 134.00]	124.00 [114.00, 135.00]	<0.05
DBP (mmHg, median [IQR])	78.00 [70.00, 87.00]	74.00 [66.00, 82.00]	75.00 [68.00, 83.00]	77.00 [69.00, 85.00]	79.00 [71.00, 87.00]	80.00 [72.00, 88.00]	81.00 [74.00, 89.00]	81.00 [74.00, 89.00]	<0.05
**Laboratory Examination**									
HR (/min, median [IQR])	70.00 [64.00, 76.00]	69.00 [64.00, 74.00]	69.00 [64.00, 75.00]	70.00 [64.00, 75.00]	70.00 [64.00, 76.00]	70.00 [64.00, 76.00]	71.00 [65.00, 76.00]	72.00 [66.00, 78.00]	<0.05
LEU (×10^9/L, median [IQR])	5.82 [4.94, 6.88]	5.48 [4.59, 6.54]	5.55 [4.70, 6.57]	5.67 [4.82, 6.68]	5.81 [4.94, 6.83]	5.94 [5.07, 6.98]	6.10 [5.24, 7.17]	6.28 [5.32, 7.41]	<0.05
RBC (×10^{12}/L, median [IQR])	4.79 [4.46, 5.12]	4.37 [4.10, 4.66]	4.51 [4.24, 4.81]	4.66 [4.37, 4.98]	4.80 [4.50, 5.10]	4.92 [4.62, 5.20]	5.05 [4.77, 5.30]	5.10 [4.82, 5.38]	<0.05
PLT (×10^9/L, median [IQR])	220.00 [188.00, 257.00]	222.00 [187.00, 259.00]	222.00 [188.00, 259.00]	221.00 [188.00, 258.00]	220.00 [187.00, 256.00]	219.00 [187.00, 255.00]	220.00 [188.00, 254.00]	224.00 [192.00, 258.00]	<0.05

HGB (g/L, median [IQR])	147.00 [135.00, 158.00]	131.00 [123.00, 141.00]	136.00 [127.00, 146.00]	142.00 [132.00, 153.00]	147.00 [137.00, 157.00]	152.00 [142.00, 160.00]	156.00 [147.00, 163.00]	157.00 [148.00, 165.00]	<0.05
ALT (IU/L, median [IQR])	19.40 [13.80, 28.70]	14.60 [11.00, 21.00]	15.60 [11.60, 22.30]	17.50 [12.70, 25.20]	19.40 [14.00, 28.00]	21.40 [15.30, 31.10]	24.00 [17.00, 35.30]	27.00 [18.70, 40.30]	<0.05
AST (IU/L, median [IQR])	18.40 [15.50, 22.60]	16.90 [14.30, 20.70]	17.10 [14.60, 20.60]	17.80 [15.10, 21.60]	18.40 [15.50, 22.40]	19.00 [16.10, 23.30]	19.90 [16.70, 24.60]	21.00 [17.30, 27.10]	<0.05
GGT (IU/L, median [IQR])	26.00 [16.00, 46.00]	16.70 [12.00, 27.20]	18.10 [12.60, 30.60]	22.00 [14.20, 38.00]	26.20 [16.60, 45.00]	30.70 [19.00, 52.00]	36.30 [23.00, 62.00]	40.20 [24.00, 70.07]	<0.05
Scr (μmol/L, median [IQR])	68.00 [57.60, 78.00]	60.15 [53.00, 69.70]	61.00 [54.00, 71.90]	64.30 [55.30, 75.80]	68.10 [58.00, 78.30]	71.00 [61.00, 80.00]	72.90 [64.00, 81.00]	72.00 [63.00, 80.00]	<0.05
BUN (mmol/L, median [IQR])	4.90 [4.11, 5.72]	4.70 [3.93, 5.66]	4.74 [4.00, 5.60]	4.80 [4.10, 5.70]	4.90 [4.14, 5.74]	4.93 [4.20, 5.79]	5.00 [4.28, 5.80]	4.98 [4.22, 5.83]	<0.05
UA (μmol/L, median [IQR])	327.00 [264.40, 391.70]	271.00 [228.00, 328.00]	284.00 [237.00, 347.00]	306.50 [250.60, 372.60]	330.00 [268.00, 393.00]	347.40 [287.10, 406.00]	363.00 [307.85, 419.00]	365.00 [307.30, 423.00]	<0.05
FBG (mmol/L, median [IQR])	5.32 [4.97, 5.82]	5.09 [4.76, 5.56]	5.19 [4.86, 5.64]	5.28 [4.94, 5.76]	5.34 [5.00, 5.83]	5.39 [5.03, 5.89]	5.42 [5.07, 5.92]	5.45 [5.09, 5.93]	<0.05
TC (mmol/L, median [IQR])	4.72 [4.15, 5.35]	4.82 [4.23, 5.51]	4.75 [4.17, 5.40]	4.74 [4.16, 5.38]	4.72 [4.15, 5.35]	4.72 [4.15, 5.32]	4.70 [4.14, 5.29]	4.61 [4.02, 5.23]	<0.05
TG (mmol/L, median [IQR])	1.37 [0.94, 2.05]	1.13 [0.80, 1.69]	1.18 [0.83, 1.76]	1.28 [0.89, 1.92]	1.38 [0.95, 2.05]	1.48 [1.02, 2.18]	1.57 [1.09, 2.30]	1.58 [1.09, 2.34]	<0.05
LDL-C (mmol/L, median [IQR])	3.00 [2.47, 3.55]	3.00 [2.45, 3.60]	2.99 [2.46, 3.55]	3.00 [2.47, 3.56]	3.00 [2.47, 3.55]	3.01 [2.49, 3.55]	3.00 [2.49, 3.52]	2.90 [2.36, 3.44]	<0.05
HDL-C (mmol/L, median [IQR])	1.24 [1.03, 1.49]	1.37 [1.13, 1.64]	1.33 [1.10, 1.60]	1.28 [1.06, 1.54]	1.23 [1.03, 1.48]	1.20 [1.00, 1.43]	1.16 [0.99, 1.38]	1.16 [0.99, 1.36]	<0.05
FT3 (pmol/L, median [IQR])	4.90 [4.48, 5.34]	3.69 [3.50, 3.80]	4.19 [4.06, 4.29]	4.57 [4.48, 4.65]	4.89 [4.81, 4.97]	5.23 [5.14, 5.33]	5.66 [5.54, 5.82]	6.29 [6.15, 6.68]	<0.05
FT4 (pmol/L, median [IQR])	16.24 [14.80, 17.80]	14.51 [13.05, 16.05]	15.23 [13.93, 16.62]	15.75 [14.44, 17.13]	16.22 [14.93, 17.60]	16.67 [15.35, 18.11]	17.26 [15.90, 18.73]	18.41 [16.69, 20.48]	<0.05
TSH (uIU/mL, median [IQR])	2.07 [1.42, 3.02]	2.42 [1.58, 3.80]	2.24 [1.53, 3.33]	2.15 [1.48, 3.15]	2.05 [1.43, 2.98]	2.00 [1.39, 2.87]	1.94 [1.36, 2.79]	1.74 [1.03, 2.61]	<0.05
**Comorbidities**									

Type 2 diabetes, n (%)	23349 (13.19)	1208 (13.85)	3444 (12.94)	4732 (13.62)	4889 (13.84)	4701 (13.23)	3390 (12.50)	985 (10.96)	<0.05
Hypertension, n (%)	44234 (26.27)	1450 (18.34)	4896 (19.75)	7796 (23.67)	9110 (26.99)	9976 (29.26)	8265 (31.54)	2741 (31.48)	<0.05
MetS, n (%)	82226 (48.46)	2946 (36.59)	9863 (39.36)	14988 (45.05)	16749 (49.25)	17990 (52.46)	14806 (56.36)	4884 (55.93)	<0.05
MAFLD, n (%)	90787 (51.14)	2811 (32.16)	10272 (38.48)	16059 (46.08)	18598 (52.46)	20534 (57.65)	17091 (62.89)	5422 (60.19)	<0.05

Abbreviations: IQR, interquartile range; BMI, body mass index; WC, waist circumference; SBP, systolic blood pressure; DBP, diastolic blood pressure; HR, heart rates; LEU, leukocyte counts; RBC, red blood cells; PLT, platelet counts; HGB, hemoglobin; ALT, alanine aminotransferase; AST, aspartate transaminase; GGT, glutamyl transpeptidase; Scr, serum creatinine; BUN, blood urea nitrogen; UA, uric acid; FBG, fasting blood glucose; TC, total cholesterol; TG, triglycerides; LDL-C, low-density lipoprotein cholesterol; HDL-C, high-density lipoprotein cholesterol; FT3, free triiodothyronine; FT4, free tetraiodothyronine; TSH, thyroid-stimulating hormone; MetS, metabolic syndrome; MAFLD, metabolic dysfunction-associated fatty liver disease.

Table S2. Clinical and laboratory characteristics of participants according to prior-defined centile categories of FT4 from 2010 to 2018.

Characteristics	All	Centile (pmol/L) 1st-5th (FT4<12.80)	6th-20th (12.80≤FT4<14.45)	21st-40th (14.45≤FT4<15.69)	41st-60th (15.69≤FT4<16.80)	61st-80th (16.80≤FT4<18.21)	81st-95th (18.21≤FT4<20.43)	96st-100th (FT4≥20.43)	P value
	N = 177540	N = 8803	N = 26528	N = 35429	N = 35513	N = 35592	N = 26788	N = 8887	
**Clinical characteristics**									
Age (years, median [IQR])	48.00 [42.00, 54.00]	50.00 [44.00, 56.00]	49.00 [43.00, 55.00]	48.00 [42.00, 54.00]	48.00 [42.00, 53.00]	47.00 [41.00, 53.00]	46.00 [40.00, 52.00]	46.00 [38.00, 52.00]	<0.05
Gender, Male, n (%)	106859 (60.19)	3201 (36.36)	11932 (44.98)	18962 (53.52)	21803 (61.39)	24351 (68.42)	19988 (74.62)	6622 (74.51)	<0.05
BMI (kg/m^2, median [IQR])	24.76 [22.48, 27.04]	24.81 [22.59, 27.15]	24.67 [22.41, 27.01]	24.72 [22.43, 27.04]	24.78 [22.49, 27.05]	24.82 [22.58, 27.08]	24.80 [22.52, 27.05]	24.60 [22.22, 26.86]	<0.05
WC (cm, median [IQR])	87.00 [79.00, 94.00]	85.00 [78.00, 93.00]	86.00 [78.00, 93.00]	87.00 [78.00, 94.00]	88.00 [79.00, 95.00]	88.00 [80.00, 95.00]	88.00 [81.00, 95.00]	87.00 [80.00, 94.00]	<0.05
Self-reported smoking, n (%)	26119 (14.71)	816 (9.27)	2962 (11.17)	4666 (13.17)	5348 (15.06)	5870 (16.49)	4861 (18.15)	1596 (17.96)	<0.05
Self-reported drinking, n (%)	39846 (22.44)	1323 (15.03)	4932 (18.59)	7327 (20.68)	8152 (22.95)	8779 (24.67)	7120 (26.58)	2213 (24.90)	<0.05
SBP (mmHg, median [IQR])	120.00 [109.00, 132.00]	119.00 [107.00, 132.00]	118.00 [107.00, 131.00]	119.00 [108.00, 132.00]	120.00 [109.00, 132.00]	121.00 [110.00, 133.00]	122.00 [111.00, 133.00]	123.00 [112.00, 134.00]	<0.05
DBP (mmHg, median [IQR])	78.00 [70.00, 87.00]	76.00 [69.00, 85.00]	77.00 [69.00, 85.00]	78.00 [70.00, 86.00]	78.00 [70.00, 87.00]	79.00 [71.00, 87.00]	80.00 [72.00, 88.00]	80.00 [72.00, 88.00]	<0.05
**Laboratory Examination**									
HR (/min, median [IQR])	70.00 [64.00, 76.00]	69.00 [64.00, 74.00]	70.00 [64.00, 75.00]	70.00 [64.00, 75.00]	70.00 [64.00, 76.00]	70.00 [64.00, 76.00]	71.00 [65.00, 76.00]	72.00 [66.00, 78.00]	<0.05
LEU (×10^9/L, median [IQR])	5.82 [4.94, 6.88]	5.61 [4.78, 6.65]	5.68 [4.81, 6.69]	5.75 [4.89, 6.79]	5.83 [4.94, 6.88]	5.89 [5.01, 6.96]	5.96 [5.07, 7.04]	6.00 [5.09, 7.12]	<0.05
RBC (×10^{12}/L, median [IQR])	4.79 [4.46, 5.12]	4.53 [4.24, 4.85]	4.62 [4.32, 4.96]	4.72 [4.40, 5.05]	4.79 [4.47, 5.11]	4.88 [4.55, 5.18]	4.95 [4.63, 5.24]	4.99 [4.67, 5.28]	<0.05
PLT (×10^9/L,	220.00	218.00	219.00	220.00	220.00	221.00	222.00	225.00	<0.05

median [IQR])	[188.00, 257.00]	[183.00, 256.00]	[185.00, 256.00]	[187.00, 257.00]	[188.00, 255.00]	[189.00, 256.00]	[191.00, 258.00]	[192.00, 261.00]	
HGB (g/L,	147.00	137.00	140.00	144.00	147.00	150.00	152.00	154.00	<0.05
median [IQR])	[135.00, 158.00]	[127.00, 149.00]	[130.00, 152.00]	[133.00, 155.00]	[135.00, 158.00]	[139.00, 159.00]	[142.00, 161.00]	[142.00, 162.00]	
ALT (IU/L,	19.40	18.20	18.30	18.90	19.40	20.00	20.20	21.00	<0.05
median [IQR])	[13.80, 28.70]	[13.10, 27.10]	[13.00, 27.30]	[13.30, 28.00]	[13.80, 28.80]	[14.00, 29.40]	[14.50, 29.60]	[15.00, 30.80]	
AST (IU/L,	18.40	18.60	18.30	18.30	18.50	18.50	18.60	18.85	<0.05
median [IQR])	[15.50, 22.60]	[15.50, 23.10]	[15.30, 22.40]	[15.40, 22.50]	[15.50, 22.60]	[15.60, 22.80]	[15.70, 22.70]	[15.70, 23.10]	
GGT (IU/L,	26.00	21.90	22.70	24.60	26.30	28.00	29.10	30.00	<0.05
median [IQR])	[16.00, 46.00]	[14.00, 39.00]	[14.10, 40.90]	[15.00, 43.80]	[16.00, 47.00]	[17.00, 49.00]	[18.40, 50.00]	[19.00, 51.00]	
Scr (μmol/L,	68.00	61.30	63.00	65.70	68.00	70.00	72.00	72.00	<0.05
median [IQR])	[57.60, 78.00]	[53.00, 72.00]	[54.20, 74.00]	[56.00, 76.00]	[58.00, 78.00]	[60.00, 79.20]	[62.00, 81.00]	[61.00, 81.20]	
BUN (mmol/L,	4.90	4.73	4.80	4.84	4.90	4.91	4.94	4.90	<0.05
median [IQR])	[4.11, 5.72]	[4.00, 5.60]	[4.02, 5.63]	[4.10, 5.70]	[4.13, 5.77]	[4.20, 5.80]	[4.20, 5.80]	[4.18, 5.80]	
UA (μmol/L,	327.00	292.80	304.00	317.00	329.80	338.60	345.00	345.30	<0.05
median [IQR])	[264.40, 391.70]	[241.00, 359.20]	[247.00, 373.00]	[256.00, 384.00]	[266.00, 393.00]	[276.00, 399.70]	[285.90, 404.00]	[286.00, 404.50]	
FBG (mmol/L,	5.32	5.25	5.28	5.30	5.33	5.35	5.37	5.38	<0.05
median [IQR])	[4.97, 5.82]	[4.90, 5.71]	[4.93, 5.75]	[4.95, 5.78]	[4.98, 5.83]	[5.00, 5.85]	[5.01, 5.88]	[5.01, 5.90]	
TC (mmol/L,	4.72	4.82	4.74	4.74	4.73	4.72	4.70	4.61	<0.05
median [IQR])	[4.15, 5.35]	[4.21, 5.50]	[4.16, 5.38]	[4.16, 5.36]	[4.16, 5.35]	[4.15, 5.33]	[4.14, 5.30]	[4.00, 5.26]	
TG (mmol/L,	1.37	1.39	1.36	1.36	1.38	1.39	1.38	1.32	<0.05
median [IQR])	[0.94, 2.05]	[0.94, 2.13]	[0.93, 2.06]	[0.93, 2.05]	[0.94, 2.07]	[0.95, 2.06]	[0.95, 2.03]	[0.93, 1.92]	
LDL-C (mmol/L,	3.00	3.01	3.00	3.01	3.00	3.01	2.99	2.90	<0.05
median [IQR])	[2.47, 3.55]	[2.47, 3.62]	[2.47, 3.56]	[2.48, 3.55]	[2.48, 3.55]	[2.48, 3.54]	[2.46, 3.53]	[2.35, 3.44]	
HDL-C (mmol/L,	1.24	1.27	1.25	1.24	1.23	1.23	1.23	1.24	<0.05
median [IQR])	[1.03, 1.49]	[1.04, 1.53]	[1.04, 1.51]	[1.03, 1.50]	[1.03, 1.49]	[1.03, 1.48]	[1.03, 1.48]	[1.05, 1.48]	
FT3 (pmol/L,	4.90	4.37	4.60	4.78	4.91	5.04	5.19	5.47	<0.05
median [IQR])	[4.48, 5.34]	[3.96, 4.78]	[4.24, 4.99]	[4.40, 5.16]	[4.52, 5.30]	[4.65, 5.44]	[4.79, 5.61]	[4.98, 6.06]	
FT4 (pmol/L,	16.24	12.15	13.81	15.11	16.23	17.43	19.00	21.46	<0.05
median [IQR])	[14.80, 17.80]	[11.49, 12.51]	[13.39, 14.15]	[14.80, 15.40]	[15.96, 16.51]	[17.10, 17.79]	[18.57, 19.55]	[20.87, 22.46]	
TSH (uIU/mL,	2.07	3.11	2.41	2.17	2.05	1.94	1.84	1.54	<0.05
median [IQR])	[1.42, 3.02]	[1.93, 5.53]	[1.63, 3.56]	[1.50, 3.16]	[1.43, 2.94]	[1.36, 2.76]	[1.29, 2.59]	[0.87, 2.31]	
**Comorbidities**									
Type 2 diabetes,	23349 (13.19)	1042 (11.87)	3222 (12.17)	4482 (12.68)	4679 (13.21)	4912 (13.84)	3767 (14.10)	1245 (14.07)	<0.05

n (%)								
Hypertension, n (%)	44234 (26.27)	1896 (23.01)	5919 (23.67)	8448 (25.18)	8807 (26.16)	9339 (27.51)	7364 (28.88)	2461 (28.95)	<0.05
MetS, n (%)	82226 (48.46)	4115 (49.45)	12185 (48.29)	16371 (48.37)	16535 (48.68)	16660 (48.79)	12383 (48.28)	3977 (46.66)	<0.05
MAFLD, n (%)	90787 (51.14)	4308 (48.94)	13362 (50.37)	18046 (50.94)	18402 (51.82)	18720 (52.60)	13748 (51.32)	4201 (47.27)	<0.05

Abbreviations: IQR, interquartile range; BMI, body mass index; WC, waist circumference; SBP, systolic blood pressure; DBP, diastolic blood pressure; HR, heart rates; LEU, leukocyte counts; RBC, red blood cells; PLT, platelet counts; HGB, hemoglobin; ALT, alanine aminotransferase; AST, aspartate transaminase; GGT, glutamyl transpeptidase; Scr, serum creatinine; BUN, blood urea nitrogen; UA, uric acid; FBG, fasting blood glucose; TC, total cholesterol; TG, triglycerides; LDL-C, low-density lipoprotein cholesterol; HDL-C, high-density lipoprotein cholesterol; FT3, free triiodothyronine; FT4, free tetraiodothyronine; TSH, thyroid-stimulating hormone; MetS, metabolic syndrome; MAFLD, metabolic dysfunction-associated fatty liver disease.

Table S3. Clinical and laboratory characteristics of participants according to prior-defined centile categories of TSH from 2010 to 2018.

Characteristics	All	Centile (uIU/mL)							P value
		1st-5th (TSH<0.75)	6th-20th (0.75≤TSH<1.29)	21st-40th (1.29≤TSH<1.80)	41st-60th (1.80≤TSH<2.38)	61st-80th (2.38≤TSH<3.33)	81st-95th (3.33≤TSH<5.60)	96st-100th (TSH≥5.60)	
	N = 177540	N = 8732	N = 26474	N = 35743	N = 35373	N = 35623	N = 26714	N = 8881	
**Clinical characteristics**									
Age (years, median [IQR])	48.00 [42.00, 54.00]	48.00 [42.00, 54.00]	48.00 [42.00, 53.00]	47.00 [41.00, 53.00]	47.00 [41.00, 53.00]	48.00 [41.00, 53.00]	48.00 [42.00, 54.00]	50.00 [43.00, 57.00]	<0.05
Gender, Male, n (%)	106859 (60.19)	5195 (59.49)	18079 (68.29)	23952 (67.01)	22222 (62.82)	20625 (57.90)	13180 (49.34)	3606 (40.60)	<0.05
BMI (kg/m^2, median [IQR])	24.76 [22.48, 27.04]	24.43 [22.15, 26.67]	24.79 [22.52, 26.99]	24.85 [22.61, 27.10]	24.89 [22.60, 27.16]	24.77 [22.46, 27.10]	24.61 [22.32, 26.95]	24.47 [22.23, 26.84]	<0.05
WC (cm, median [IQR])	87.00 [79.00, 94.00]	87.00 [78.00, 94.00]	88.00 [80.00, 95.00]	88.00 [80.00, 95.00]	88.00 [80.00, 95.00]	87.00 [79.00, 94.00]	86.00 [78.00, 93.00]	85.00 [77.00, 92.00]	<0.05
Self-reported smoking, n (%)	26119 (14.71)	1383 (15.84)	4923 (18.60)	6283 (17.58)	5447 (15.40)	4666 (13.10)	2705 (10.13)	712 (8.02)	<0.05
Self-reported drinking, n (%)	39846 (22.44)	1760 (20.16)	6562 (24.79)	8949 (25.04)	8468 (23.94)	7894 (22.16)	4949 (18.53)	1264 (14.23)	<0.05
SBP (mmHg, median [IQR])	120.00 [109.00, 132.00]	120.00 [109.00, 132.00]	120.00 [109.00, 132.00]	120.00 [109.00, 132.00]	120.00 [109.00, 132.00]	120.00 [109.00, 133.00]	120.00 [109.00, 133.00]	121.00 [109.00, 134.00]	<0.05
DBP (mmHg, median [IQR])	78.00 [70.00, 87.00]	77.00 [70.00, 85.00]	78.00 [70.00, 86.00]	78.00 [71.00, 87.00]	79.00 [71.00, 87.00]	78.00 [71.00, 87.00]	78.00 [71.00, 87.00]	78.00 [70.00, 86.00]	<0.05
**Laboratory Examination**									
HR (/min, median [IQR])	70.00 [64.00, 76.00]	71.00 [65.00, 76.00]	70.00 [65.00, 76.00]	70.00 [64.00, 76.00]	70.00 [64.00, 76.00]	70.00 [64.00, 76.00]	70.00 [64.00, 76.00]	70.00 [64.00, 76.00]	<0.05
LEU (×10^9/L, median [IQR])	5.82 [4.94, 6.88]	5.89 [4.96, 7.01]	5.89 [4.97, 6.99]	5.85 [4.98, 6.91]	5.81 [4.95, 6.85]	5.80 [4.92, 6.83]	5.78 [4.90, 6.79]	5.74 [4.87, 6.80]	<0.05
RBC (×10^{12}/L, median [IQR])	4.79 [4.46, 5.12]	4.78 [4.46, 5.11]	4.84 [4.51, 5.15]	4.84 [4.50, 5.15]	4.82 [4.48, 5.14]	4.78 [4.44, 5.11]	4.71 [4.40, 5.06]	4.64 [4.34, 4.97]	<0.05
PLT (×10^9/L, median [IQR])	220.00 [188.00, 257.00]	220.00 [187.00, 257.00]	220.00 [188.00, 255.00]	220.00 [188.00, 256.00]	220.00 [188.00, 256.00]	221.00 [188.00, 257.00]	221.00 [188.00, 258.00]	221.00 [186.00, 259.00]	0.10

HGB (g/L, median [IQR])	147.00 [135.00, 158.00]	146.00 [134.00, 157.00]	149.00 [137.00, 159.00]	149.00 [137.00, 159.00]	148.00 [136.00, 158.00]	146.00 [134.00, 157.00]	143.00 [133.00, 155.00]	140.00 [131.00, 152.00]	<0.05
ALT (IU/L, median [IQR])	19.40 [13.80, 28.70]	19.70 [13.90, 28.60]	19.50 [13.98, 28.60]	19.70 [14.00, 29.00]	19.70 [13.90, 29.00]	19.30 [13.60, 29.00]	18.80 [13.40, 28.00]	18.40 [13.30, 27.00]	<0.05
AST (IU/L, median [IQR])	18.40 [15.50, 22.60]	17.90 [15.10, 22.00]	18.10 [15.30, 22.20]	18.30 [15.40, 22.50]	18.40 [15.60, 22.60]	18.60 [15.60, 22.80]	18.70 [15.70, 23.00]	19.00 [16.00, 23.50]	<0.05
GGT (IU/L, median [IQR])	26.00 [16.00, 46.00]	26.60 [17.00, 46.00]	28.00 [17.00, 48.50]	27.40 [17.00, 48.00]	27.00 [16.10, 47.00]	25.70 [15.80, 45.70]	24.00 [15.00, 42.90]	22.00 [14.20, 39.00]	<0.05
Scr (µmol/L, median [IQR])	68.00 [57.60, 78.00]	65.00 [55.00, 75.23]	68.80 [58.50, 78.00]	69.00 [59.00, 78.60]	68.50 [58.00, 78.10]	67.60 [57.00, 78.00]	66.00 [56.40, 77.00]	65.00 [56.00, 76.30]	<0.05
BUN (mmol/L, median [IQR])	4.90 [4.11, 5.72]	4.90 [4.10, 5.80]	4.92 [4.20, 5.80]	4.90 [4.18, 5.80]	4.90 [4.11, 5.72]	4.82 [4.10, 5.70]	4.80 [4.10, 5.70]	4.82 [4.08, 5.70]	<0.05
UA (µmol/L, median [IQR])	327.00 [264.40, 391.70]	319.00 [261.00, 383.00]	333.80 [272.00, 394.00]	335.00 [271.83, 396.30]	332.00 [267.22, 396.00]	326.00 [263.00, 392.00]	314.65 [255.00, 383.00]	301.60 [248.80, 368.00]	<0.05
FBG (mmol/L, median [IQR])	5.32 [4.97, 5.82]	5.36 [4.99, 5.87]	5.35 [5.00, 5.87]	5.34 [4.99, 5.84]	5.33 [4.98, 5.82]	5.31 [4.96, 5.80]	5.29 [4.94, 5.77]	5.26 [4.90, 5.71]	<0.05
TC (mmol/L, median [IQR])	4.72 [4.15, 5.35]	4.56 [3.98, 5.18]	4.68 [4.12, 5.29]	4.70 [4.13, 5.31]	4.73 [4.15, 5.34]	4.74 [4.17, 5.36]	4.79 [4.20, 5.41]	4.88 [4.27, 5.57]	<0.05
TG (mmol/L, median [IQR])	1.37 [0.94, 2.05]	1.28 [0.90, 1.89]	1.34 [0.92, 2.01]	1.38 [0.94, 2.07]	1.39 [0.95, 2.09]	1.38 [0.94, 2.07]	1.38 [0.95, 2.05]	1.36 [0.95, 2.02]	<0.05
LDL-C (mmol/L, median [IQR])	3.00 [2.47, 3.55]	2.86 [2.33, 3.42]	2.98 [2.46, 3.52]	2.99 [2.46, 3.53]	3.01 [2.48, 3.55]	3.00 [2.47, 3.55]	3.03 [2.50, 3.58]	3.06 [2.53, 3.66]	<0.05
HDL-C (mmol/L, median [IQR])	1.24 [1.03, 1.49]	1.23 [1.02, 1.49]	1.22 [1.01, 1.47]	1.22 [1.02, 1.47]	1.23 [1.03, 1.48]	1.25 [1.04, 1.50]	1.27 [1.05, 1.52]	1.31 [1.08, 1.57]	<0.05
FT3 (pmol/L, median [IQR])	4.90 [4.48, 5.34]	5.13 [4.63, 5.76]	4.96 [4.55, 5.39]	4.94 [4.53, 5.37]	4.92 [4.51, 5.35]	4.88 [4.46, 5.31]	4.80 [4.39, 5.23]	4.63 [4.21, 5.09]	<0.05
FT4 (pmol/L, median [IQR])	16.24 [14.80, 17.80]	17.50 [15.72, 19.96]	16.66 [15.29, 18.20]	16.49 [15.14, 17.99]	16.32 [14.95, 17.83]	16.12 [14.72, 17.60]	15.67 [14.29, 17.15]	14.51 [12.84, 16.09]	<0.05
TSH (uIU/mL, median [IQR])	2.07 [1.42, 3.02]	0.53 [0.23, 0.66]	1.07 [0.93, 1.18]	1.54 [1.42, 1.67]	2.07 [1.93, 2.21]	2.77 [2.56, 3.02]	4.02 [3.62, 4.60]	7.17 [6.19, 9.40]	<0.05
**Comorbidities**									

Type 2 diabetes, n (%)	23349 (13.19)	1280 (14.72)	3857 (14.61)	4845 (13.59)	4655 (13.19)	4457 (12.55)	3211 (12.06)	1044 (11.80)	<0.05
Hypertension, n (%)	44234 (26.27)	1987 (24.31)	6352 (25.45)	8840 (26.12)	9007 (26.78)	8995 (26.51)	6745 (26.51)	2308 (27.40)	<0.05
MetS, n (%)	82226 (48.46)	3897 (47.33)	12191 (48.38)	16773 (49.07)	16656 (49.18)	16462 (48.21)	12278 (47.96)	3969 (46.94)	<0.05
MAFLD, n (%)	90787 (51.14)	4126 (47.25)	13715 (51.81)	18899 (52.87)	18731 (52.95)	18111 (50.84)	13185 (49.36)	4020 (45.27)	<0.05

Abbreviations: IQR, interquartile range; BMI, body mass index; WC, waist circumference; SBP, systolic blood pressure; DBP, diastolic blood pressure; HR, heart rates; LEU, leukocyte counts; RBC, red blood cells; PLT, platelet counts; HGB, hemoglobin; ALT, alanine aminotransferase; AST, aspartate transaminase; GGT, glutamyl transpeptidase; Scr, serum creatinine; BUN, blood urea nitrogen; UA, uric acid; FBG, fasting blood glucose; TC, total cholesterol; TG, triglycerides; LDL-C, low-density lipoprotein cholesterol; HDL-C, high-density lipoprotein cholesterol; FT3, free triiodothyronine; FT4, free tetraiodothyronine; TSH, thyroid-stimulating hormone; MetS, metabolic syndrome; MAFLD, metabolic dysfunction-associated fatty liver disease.

Table S4. Clinical and laboratory characteristics of participants with and without MAFLD from 2010 to 2017 in sensitivity analysis 2.

**Characteristics**	Total (N = 9437)	Non-MAFLD (N = 6696, 70.95%)	MAFLD (N = 2741, 20.05%)	P-value
**Clinical characteristics**				
Age (years, median [IQR])	45.00 [37.00, 53.00]	45.00 [35.00, 53.00]	47.00 [39.00, 54.00]	<0.05
Gender, Male, n (%)	5282 (55.97)	3163 (47.24)	2119 (77.31)	<0.05
BMI (kg/m^2, median [IQR])	23.76 [21.56, 26.02]	22.54 [20.82, 24.45]	26.43 [24.79, 28.33]	<0.05
WC (cm, median [IQR])	82.00 [75.00, 90.00]	80.00 [72.00, 85.00]	91.00 [86.00, 96.00]	<0.05
Self-reported smoking, n (%)	-	-	-	-
Self-reported drinking, n (%)	-	-	-	-
SBP (mmHg, median [IQR])	120.00 [110.00, 133.00]	118.00 [108.00, 130.00]	128.00 [118.00, 138.00]	<0.05
DBP (mmHg, median [IQR])	74.00 [65.00, 80.00]	70.00 [64.00, 78.00]	78.00 [72.00, 86.00]	<0.05
**Laboratory Examination**				
HR (/min, median [IQR])	72.00 [70.00, 76.00]	72.00 [70.00, 76.00]	72.00 [68.00, 76.00]	0.08
LEU ($\times10^9$/L, median [IQR])	6.10 [5.20, 7.27]	5.91 [5.10, 7.00]	6.70 [5.70, 7.80]	<0.05
RBC ($\times10^{12}$/L, median [IQR])	4.81 [4.45, 5.18]	4.70 [4.37, 5.07]	5.06 [4.76, 5.35]	<0.05
PLT ($\times10^9$/L, median [IQR])	224.00 [191.00, 261.00]	222.00 [188.00, 258.00]	230.00 [196.00, 269.00]	<0.05
HGB (g/L, median [IQR])	146.00 [134.00, 157.00]	142.00 [132.00, 154.00]	154.00 [144.00, 162.00]	<0.05
ALT (IU/L, median [IQR])	20.00 [15.00, 29.00]	18.00 [13.20, 24.00]	29.00 [21.00, 41.00]	<0.05
AST (IU/L, median [IQR])	22.00 [18.00, 26.00]	21.00 [18.00, 25.00]	24.00 [21.00, 30.00]	<0.05
GGT (IU/L, median [IQR])	21.00 [14.00, 35.00]	18.00 [12.00, 28.00]	32.00 [23.00, 51.00]	<0.05
Scr (μmol/L, median [IQR])	66.00 [56.00, 77.00]	64.00 [55.00, 75.79]	71.13 [62.00, 80.00]	<0.05
BUN (mmol/L, median [IQR])	4.89 [4.10, 5.72]	4.80 [4.02, 5.68]	5.04 [4.33, 5.89]	<0.05
UA (μmol/L, median [IQR])	328.00 [266.00, 397.00]	305.00 [252.00, 370.00]	384.00 [326.00, 444.85]	<0.05
FBG (mmol/L, median [IQR])	4.98 [4.67, 5.36]	4.91 [4.62, 5.25]	5.21 [4.84, 5.76]	<0.05
TC (mmol/L, median [IQR])	4.67 [4.11, 5.26]	4.57 [4.03, 5.15]	4.89 [4.34, 5.53]	<0.05
TG (mmol/L, median [IQR])	1.24 [0.85, 1.84]	1.07 [0.77, 1.49]	1.86 [1.36, 2.64]	<0.05
LDL-C (mmol/L, median [IQR])	2.71 [2.24, 3.23]	2.64 [2.18, 3.14]	2.91 [2.45, 3.49]	<0.05
HDL-C (mmol/L, median [IQR])	1.20 [1.02, 1.42]	1.26 [1.07, 1.49]	1.05 [0.93, 1.20]	<0.05
FT3 (pg/L, median [IQR])	3.22 [2.95, 3.49]	3.17 [2.92, 3.44]	3.34 [3.08, 3.60]	<0.05
FT4 (ng/dL, median [IQR])	1.14 [0.99, 1.27]	1.14 [1.00, 1.27]	1.13 [0.97, 1.27]	<0.05
TSH (uIU/mL, median [IQR])	1.92 [1.37, 2.78]	1.92 [1.37, 2.80]	1.92 [1.36, 2.76]	0.55
**Comorbidities**				
Type 2 diabetes, n (%)	649 (6.88)	261 (3.90)	388 (14.16)	<0.05
Hypertension, n (%)	2163 (23.29)	1171 (17.86)	992 (36.31)	<0.05
MetS, n (%)	3063 (34.81)	1296 (20.72)	1767 (69.43)	<0.05

Abbreviations: MAFLD, metabolic dysfunction-associated fatty liver disease; IQR, interquartile range; BMI, body mass index; WC, waist circumference; SBP, systolic blood pressure; DBP, diastolic blood pressure; HR, heart rates; LEU, leukocyte counts; RBC, red blood cells; PLT, platelet counts; HGB, hemoglobin; ALT, alanine aminotransferase; AST, aspartate transaminase; GGT, gamma-glutamyl transpeptidase; Scr, serum creatinine; BUN, blood urea nitrogen; UA, uric acid; FBG, fasting blood glucose; TC, total cholesterol; TG, triglycerides; LDL-C, low-density lipoprotein cholesterol; HDL-C, high-density lipoprotein cholesterol; FT3, free triiodothyronine; FT4, free tetraiodothyronine; TSH, thyroid-stimulating hormone; MetS, metabolic syndrome.

Figure S1

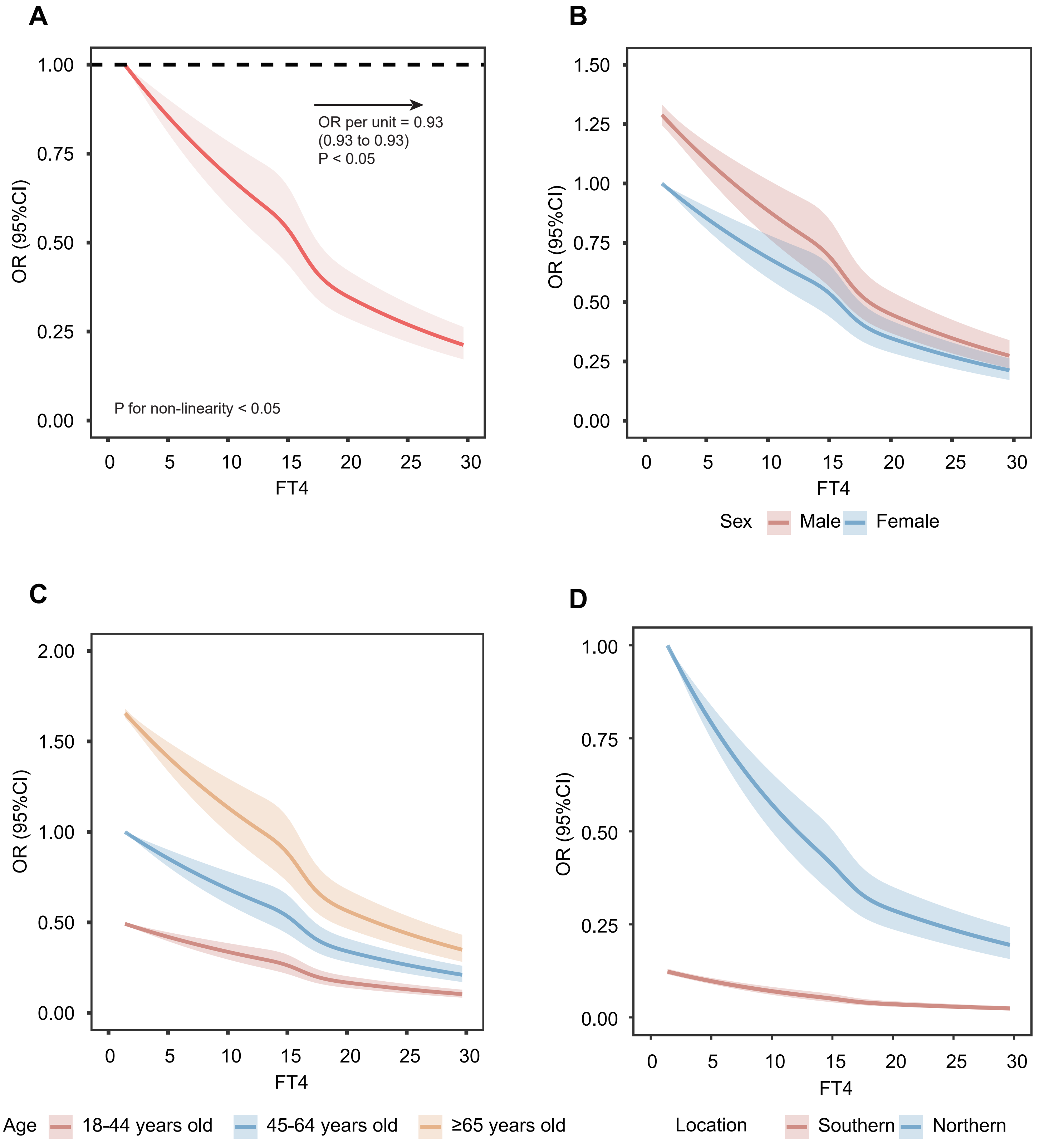

Figure S1. Restricted cubic spline analyses with four knots for nonlinear association between FT4 levels and MAFLD on a continuous scale. (A) all population. (B) by sex. (C) by age groups. (D) by location groups. Odd ratios are indicated by solid lines and 95% CIs by shaded areas. Reference point is lowest value for FT4. Analyses were adjusted for age, sex, heart rates, leukocyte counts, red blood cells, platelets, hemoglobin, gamma-glutamyl transpeptidase, estimated glomerular filtration rate, uric acid, total cholesterol, smoking status, alcohol consumption, and a history of diabetes and hypertension. FT4, free tetraiodothyronine; MAFLD, metabolic dysfunction-associated fatty liver disease; OR, odd ratio; CI, confidence interval.

Figure S2

Centile	FT4 (pmol/L)	Individuals	MAFLD	OR (95% CI)	P value
1st−5th	FT4<12.80	8803	4308	Reference	
6th−20th	12.80≤FT4<14.45	26528	13362	0.93 (0.88,0.99)	<0.05
21st−40th	14.45≤FT4<15.69	35429	18046	0.81 (0.77,0.86)	<0.05
41st−60th	15.69≤FT4<16.80	35513	18402	0.74 (0.70,0.78)	<0.05
61st−80th	16.80≤FT4<18.21	35592	18720	0.67 (0.63,0.71)	<0.05
81st−95th	18.21≤FT4<20.43	26788	13748	0.57 (0.54,0.60)	<0.05
96th−100th	FT4≥20.43	8887	4201	0.45 (0.42,0.48)	<0.05

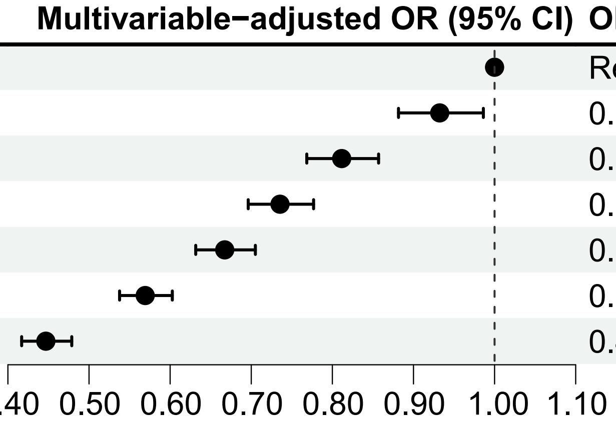

Figure S2. Multivariable adjusted logistic regression analyses for MAFLD according to FT4 levels by the prior-defined centile categories. Analyses were adjusted for age, sex, heart rates, leukocyte counts, red blood cells, platelets, hemoglobin, gamma-glutamyl transpeptidase, estimated glomerular filtration rate, uric acid, total cholesterol, smoking status, alcohol consumption, and a history of diabetes and hypertension. MAFLD, metabolic dysfunction-associated fatty liver disease; OR, odd ratio; FT4, free tetraiodothyronine.

Figure S3

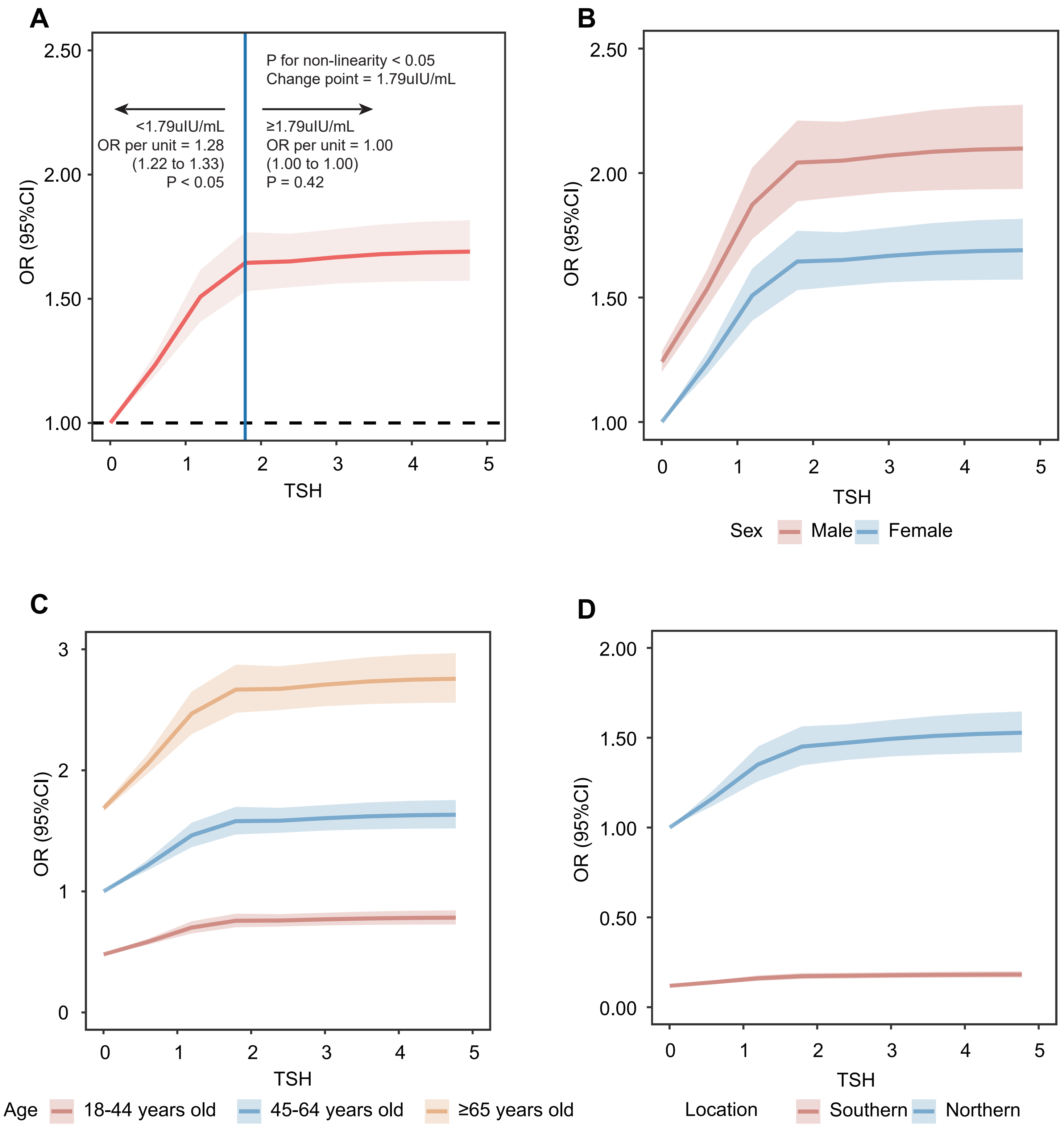

Figure S3. Restricted cubic spline analyses with five knots for nonlinear association between TSH levels and MAFLD on a continuous scale. (A) all population. (B) by sex. (C) by age groups. (D) by location groups. ORs are indicated by solid lines and 95% CIs by shaded areas. Reference point is lowest value for TSH. Analyses were adjusted for age, sex, heart rates, leukocyte counts, red blood cells, platelets, hemoglobin, gamma-glutamyl transpeptidase, estimated glomerular filtration rate, uric acid, total cholesterol, smoking status, alcohol consumption, and a history of diabetes and hypertension. TSH, thyroid stimulating hormone; MAFLD, metabolic dysfunction-associated fatty liver disease; OR, odd ratio; CI, confidence interval.

Figure S4

Centile	TSH (uIU/mL)	Individuals	MAFLD	Multivariable−adjusted OR (95% CI)	OR (95% CI)	P value
1st−5th	TSH<0.75	8732	4126		Reference	
6th−20th	0.75≤TSH<1.29	26474	13715		1.17 (1.11,1.24)	<0.05
21st−40th	1.29≤TSH<1.80	35743	18899		1.28 (1.21,1.35)	<0.05
41st−60th	1.80≤TSH<2.38	35373	18731		1.37 (1.30,1.45)	<0.05
61st−80th	2.38≤TSH<3.33	35623	18111		1.33 (1.26,1.41)	<0.05
81st−95th	3.33≤TSH<5.60	26714	13185		1.40 (1.33,1.48)	<0.05
96th−100th	TSH≥5.60	8881	4020		1.30 (1.21,1.39)	<0.05

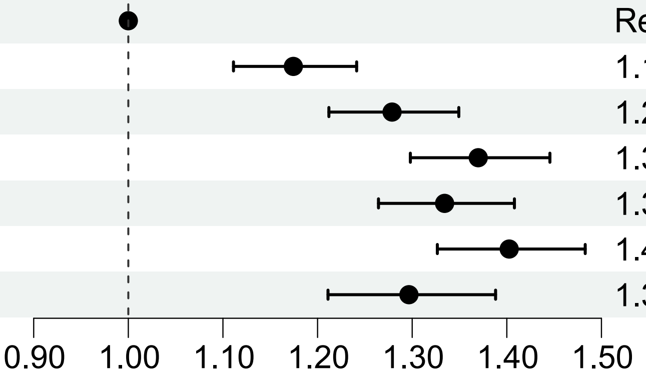

Figure S4. Multivariable adjusted logistic regression analyses for MAFLD according to TSH levels by the prior-defined centile categories. Analyses were adjusted for age, sex, heart rates, leukocyte counts, red blood cells, platelets, hemoglobin, gamma-glutamyl transpeptidase, estimated glomerular filtration rate, uric acid, total cholesterol, smoking status, alcohol consumption, and a history of diabetes and hypertension. MAFLD, metabolic dysfunction-associated fatty liver disease; TSH, thyroid stimulating hormone; OR, odd ratio; CI, confidence interval.

Figure S5

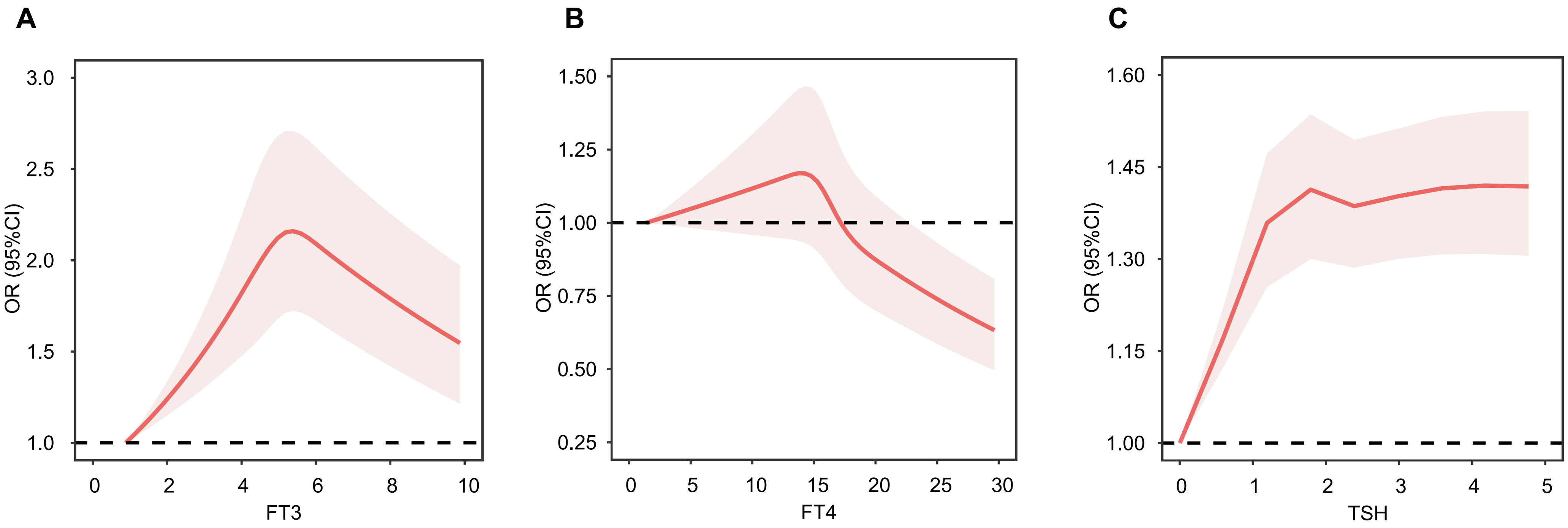

Figure S5. Restricted cubic spline analyses with different knots for nonlinear association between thyroid function parameters and MAFLD on a continuous scale in sensitivity analysis 1. (A) FT3. (B) FT4. (C) TSH. ORs are indicated by solid lines and 95% CIs by shaded areas. Reference point is lowest value for thyroid function parameters. Analyses were adjusted for age, sex, BMI, heart rates, leukocyte counts, red blood cells, platelets, hemoglobin, gamma-glutamyl transpeptidase, estimated glomerular filtration rate, uric acid, total cholesterol, smoking status, alcohol consumption, and a history of diabetes and hypertension. MAFLD, metabolic dysfunction-associated fatty liver disease; FT3, free triiodothyronine; FT4, free tetraiodothyronine, TSH, thyroid stimulating hormone; OR, odd ratio; CI, confidence interval; BMI, body mass index.

Figure S6

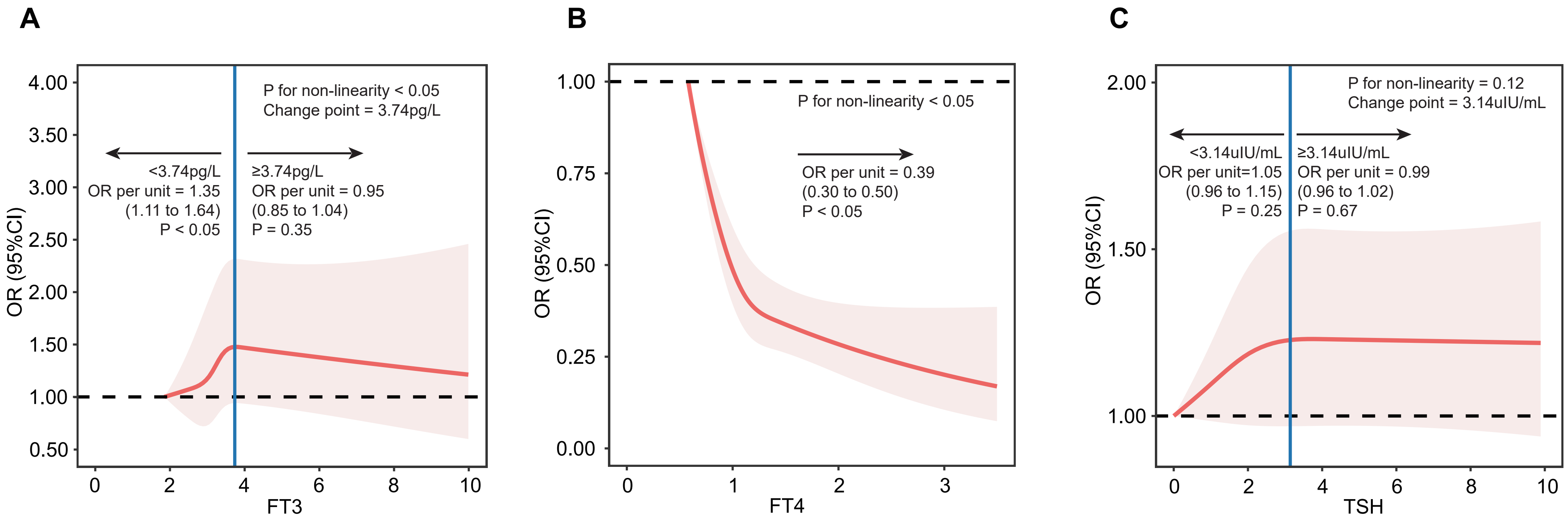

Figure S6. Restricted cubic spline analyses with different knots for nonlinear association between thyroid function parameters and MAFLD on a continuous scale in sensitivity analysis 2. (A) FT3. (B) FT4. (C) TSH. ORs are indicated by solid lines and 95% CIs by shaded areas. Reference point is lowest value for thyroid function parameters. Analyses were adjusted for age, sex, BMI, heart rates, leukocyte counts, red blood cells, platelets, hemoglobin, gamma-glutamyl transpeptidase, estimated glomerular filtration rate, uric acid, total cholesterol, and a history of diabetes and hypertension. MAFLD, metabolic dysfunction-associated fatty liver disease; FT3, free triiodothyronine; FT4, free tetraiodothyronine, TSH, thyroid stimulating hormone; OR, odd ratio; CI, confidence interval; BMI, body mass index.
